# The impact of transportation on wounds up to 4 cm on umbilical outpouchings of slaughter pigs

**DOI:** 10.1186/s13028-025-00834-5

**Published:** 2025-11-28

**Authors:** Tina Birk Jensen, Benjamin Meyer Jørgensen, Christoffer Kirkelund Flyger, Andreas Birch, Jeanett Snitgaard Pelck, Niels-Peder Nielsen, Henrik Elvang Jensen

**Affiliations:** 1 Danish Agriculture and Food Council, Axelborg, Vesterbrogade 4A, 1620 Copenhagen V, Denmark; 2 Department of Veterinary and Animal Sciences, Section for Pathobiological Sciences, University of Copenhagen, Grønnegårdsvej 15, 1870 Frederiksberg C, Denmark; 3 Ø-Vet, Danish Pig Veterinary Practice, Køberupvej 33, 4700 Næstved, Denmark; 4 Livestock Innovation, Statistics and Analyses, SEGES Innovation, Agro Food Park 15, 8200 Aarhus, Denmark

**Keywords:** Clinical signs, Fitness for transport, Necropsy, Pigs, Umbilical outpouchings, Wounds

## Abstract

**Background:**

In Denmark, pigs with wounds on umbilical outpouchings (UO) are deemed unfit for transport, due to welfare concerns, particularly the risk of wound rupture when these pigs are transported. Consequently, these pigs are often killed on-farm thereby affecting sustainability of pork production and farmers’ economy. To gain more knowledge on the impact of transportation on wounds on UOs of pigs, this study examined 96 Danish slaughter pigs with wounds on the UOs and investigated how clinical signs were associated with the distance between the wound surface and the cavity of the UOs measured at necropsy. The pigs originated from three conventional herds and were clinically examined by the herd veterinarian the day before transport to the abattoir. From the abattoir, the UOs were sent for pathological examination at University of Copenhagen where the distance between the wound surface and the peritoneal lining of the cavity of the UOs was measured.

**Results:**

The results showed that the maximum measurement of either the length or width of the wound measured clinically was 2.93 cm (σ = 0.97). The mean distance between the wound surface and the cavity of the UOs of 87 slaughter pigs was 2.27 cm, and a total of 94% had a distance of at least 1 cm. For one pig there was access from the UO wound to the UO cavity. The association between size of the wound and the distance between the wound surface and the UO cavity, depended on the shape of the UO. If the UO was elongated in shape no significant association was found, however, if the UO was spherical in shape the distance became significantly shorter as the wound became larger. The study also investigated if the wounds on the UOs changed in size when measured before and after transport to the abattoir. Wound size measured clinically and at necropsy showed a correlation of 0.51, indicating a moderate correlation. When comparing the wound sizes clinically and just after individual slaughter the correlation was 0.7 indicating a moderate to strong correlation. With a ± 10% difference from the baseline, a total of 28 wounds measured larger at the abattoir compared to the clinical examination.

**Conclusions:**

This study found that 94% of the wounds on UOs of 87 slaughter pigs examined post mortem had a distance of at least 1 cm from the wound surface to the UO cavity. In one pig the wound had ruptured giving access from the UO wound to the UO cavity. While a number of other factors also need to be considered when evaluating fitness for transport, the results from this study may serve as part of a risk assessment for transporting slaughter pigs with wounds on UOs in the future.

## Background

Pigs with umbilical outpouchings (UOs) cause a problem for the Danish pig production. A new Danish study found the prevalence of weaners with UOs to be nearly three percent [1]. The main pathological manifestations of UOs are umbilical hernias, enterocystomas and abscesses—and often combinations of these are seen [1, 2]. Pigs with UOs are typically fattened for slaughter and must be transported to an abattoir.

According to regulations from the EU, there are restrictions to when pigs are fit for transport. In general, pigs that are not able to move or pigs with severe open wounds are not fit for transport [3]. A previous study has found that the presence of an UO may affect the animal welfare and behavior of the pigs [4]. Reduced daily weight gain has been reported in pigs with UOs [5]. Although it only occurs occasionally, the most serious potential consequence regarding animal welfare is strangulation of the intestines within the UO [6]. Pigs with UOs are at risk of getting a wound on the UO—and often the wounds are placed ventrally on the UO, which has been speculated to be caused by abrasions from the floor when the UOs are touching the floor [6, 7]. In a publication examining the same pigs as in this study, we reported that 72% of 96 pigs with wounded UOs transported to slaughter had a number of acute and chronic intra-abdominal lesions found at necropsy [8].

Based on a veterinary expert statement, the Danish transport regulations require pigs to be placed in a separate compartment of the lorry with extra space and soft bedding if the UOs are greater than 15 cm in diameter [9]. If the veterinarian completes a certificate, it is possible to transport up to 5 pigs with UO in each of the separated compartments on the lorry [9]. The Danish guidelines ban transport of pigs with wounds on the UOs—regardless of the severity of the wounds [9]. The main reason is that transportation can be challenging for these pigs and cause a possible risk of the wound to rupture during transport leading to intestinal prolapse [10].

Consequently, Danish pigs with wounded UOs are killed in the herds just before slaughter regardless of the size and severity of the wound. These pigs have taken up space and consumed feed without being converted into meat, and thus these pigs might affect the sustainability of pork production and farmers’ economy. Given the high prevalence of pigs with UOs in Danish herds [1], more knowledge is needed on wounds on UOs of pigs in relation to the risk of transportation.

The objective of this study was to examine the clinical and pathological characteristics of wounds on UOs of pigs close to slaughter, and to investigate if clinical signs recorded before transport were associated with alterations in the pathological characteristics of the wounds. Finally, the study investigated if the wounds on the UOs changed in size when measured before and after a short-term transportation to the abattoir.

## Methods

The Danish Animal Experiments Inspectorate gave permission to transport a maximum of 100 slaughter pigs with wounds on the UOs (permission number 2022-15-0201-01214). The study was carried out from November 2022 to June 2023. Pigs with wounds on the UOs from three Danish conventional pig herds were included in the study. The herds were selected based on their willingness to participate, the ability of the same veterinarian to clinically examine the animals, and the requirement of the ethical permission for transport time to the abattoir not to exceed two hours. A transport time of maximum two hours from the herd to the abattoir was defined as a short term transportation. The ethical permission required that for pigs to be included, the UOs were not allowed to be clinically hard and distended. Furthermore, the ethical permission did not allow the UOs to be hot or associated with pain, and the wounds had to be dry and less than 4 cm in diameter. The clinical sign “hot” was evaluated by palpating the UO and comparing its temperature to the surrounding skin. The clinical sign “pain” was assessed based on the pig's physical reaction when palpating the UO. Pain was identified by abrupt movements or extensive vocalization when palpating the UO.

The UO’s were examined at three different times: (1) clinically, one day before transport while the pig was still alive on the farm; (2) on the day of transport, immediately after being killed at the abattoir; and (3) after the slaughter process, when the UOs were sent for pathological examination at the University of Copenhagen.

All pigs were clinically inspected the day before transportation to the abattoir by a veterinary practitioner, and during the clinical examination, the pigs were restrained by a snare. A clinical protocol was made and evaluated by other veterinarians and experts prior to the study in order to ensure an exact and transparent clinical evaluation of pigs. The diameter of the wound was assessed by measuring the maximum of the length and width of the wound using a ruler (cm). The UOs were classified as either spherical (mainly round in shape) or elongated (mainly oval in shape). Clinical examinations involved palpating the UO to determine if its content was reducible meaning that the content of the UO could be manipulated back into the abdominal cavity. The location of the wound on the UO was inspected and recorded as either ventral on the UO or not, based on whether most of the wound was situated ventrally on the UO. Wounds that were not placed ventrally on the UO were either placed caudally, cranially or on the left or right side of the UO. It was evaluated whether the wounds were clinically superficial or deep. A superficial wound was defined as a wound that only showed loss of epidermis, whereas a wound defined as deep showed loss of the dermis and the subcutaneous tissue. For each wound it was evaluated if the wound was freely manipulable over the underlying tissues (yes, no) and if granulation tissue was present (yes, no). A picture of the UO wound (together with the ruler) was taken during the clinical examination. The following day a maximum of seven pigs were transported for a maximum of two hours to the abattoir in special compartments on the lorry with extra space and extra thick layer of straw. Upon arrival at the abattoir, the pigs were placed in a separation pen and killed individually by staff using captive bolt stunning followed by bleeding. The carcasses were hung up by the hind legs on hangers, and the wounds were measured using a ruler (cm) and pictures were taken. After the slaughter process, the UOs and the intestines were sent to University of Copenhagen for pathological examination. The intra-abdominal lesions in the animals were recently presented elsewhere [8]. During necropsy, the pathological characterization of the UOs were recorded and the length and width of the wounds were measured (cm). The distance between the wound surface and the UO cavity was measured using a ruler (cm). All macroscopic measurements at necropsy were recorded in a standardized manner on a photo-table with rulers. A histological evaluation was performed for each wound. Full-size samples of the wounds were fixated in 10% neutral buffered formalin for 14 days and embedded in paraffin. Sections of 3 µm were stained with haematoxylin–eosin, scanned (ZEISS AxioScan 7, ZEISS microscopy, Germany) and uploaded to QuPath version 0.5.1 [11] for determination of the thickness (in µm) of the scab, superficial wound-associated granulation tissue (S-GT) and profound fibrotic tissue (P-FT) in relation to the wall of the hernia/enterocystoma. The scab was defined as the top layer with cellular infiltrations and necrosis. In relation to the wound surface, the S-GT was identified from perpendicular running blood vessels, whereas the P-FT contained parallel running blood vessels.

### Statistical analyses

A multivariable hierarchical model was made to assess the association between the clinical signs recorded in the herds and the distance between the surface of the wound and the UO cavity measured at necropsy. The statistical software system R version 4.2.2. was used. To allow for variation between the transport dates, we included transport date as random effects in the model.

An initial model for each analysis was found using lasso regression and best subset selection to determine which clinical signs (fixed effects) and biological plausible interactions of clinical signs that should be further examined.

To reduce the fixed effects in the initial model, we used a backwards elimination strategy using a significance level of 5% to exclude factors. To assess the sizes of the wounds measured in the herd, at the abattoir and at necropsy Pearson’s correlations coefficients were calculated.

## Results

**Fig. 1:**
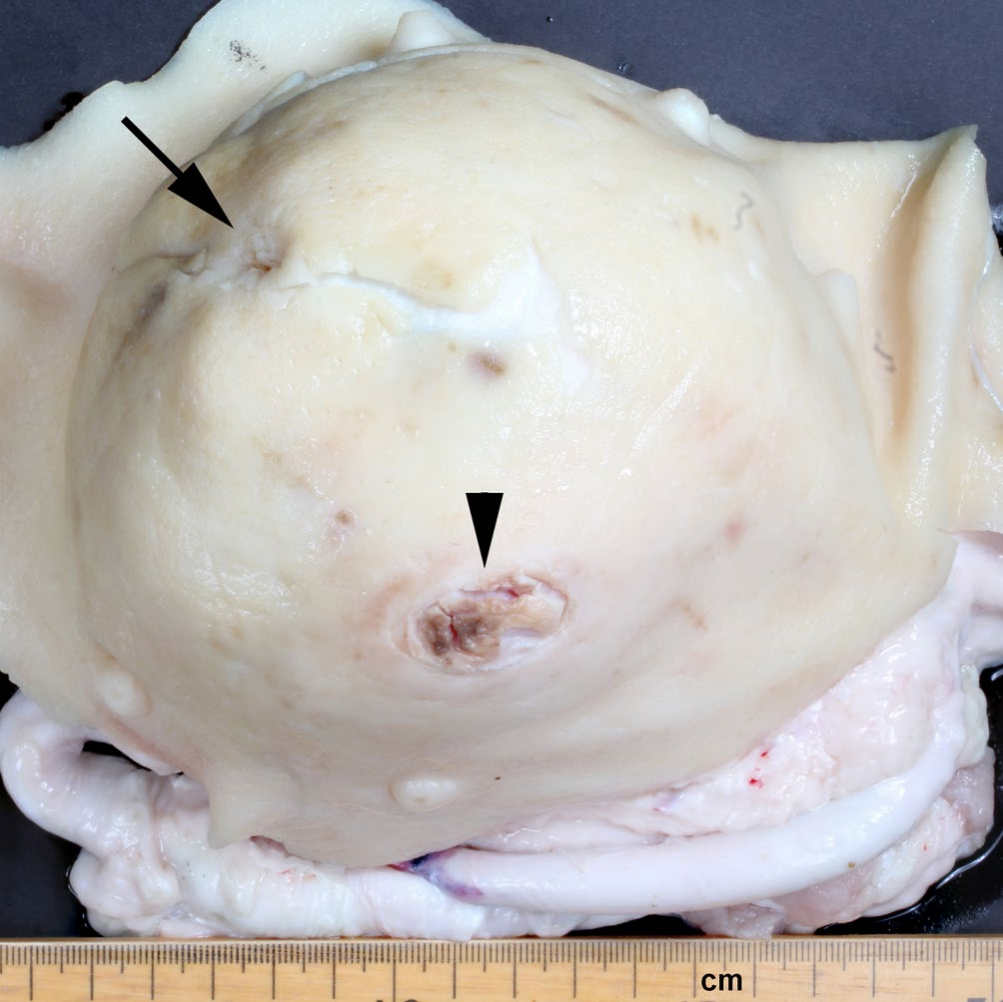
Wound on an umbilical outpouching. Umbilical outpouching from a pig with a ventrally located wound (arrowhead), caudo-ventrally to the preputial opening (arrow)

A total of 21 transports of pigs with UOs were carried out, and 96 slaughter pigs had recordings of both clinical signs and pathological observations. The journeys were equally distributed between the three herds. One pig had a wound length of 6.5 cm (special permission was granted for this pig). We chose to exclude this observation from the analyses, as there were no other data points between the maximum of 4 cm and the observation of 6.5 cm. There was a strong correlation between the area of the wound (measured as length times width regardless of the shape of the wound) and the maximum measurement of either the length or width of the wound (r = 0.83). The maximum measurement of either the length or width of the wound was therefore used as a measurement of wound size in this study. A total of 42 UOs (44%) were clinically described as elongated in shape and 53 UOs (56%) were described as spherical. A total of 58 UOs (61%) were reducible. Most wounds were located ventrally (85%) and were clinically recorded as superficial (84%) (Fig. 1).

Moreover, most wounds were freely manipulable over the underlying tissue (83%) and granulation tissue was present in 80% of the wounds (Table 1).

**Table 1:** Descriptive statistics of clinical and pathological recordings in 95 pigs

Recordings	Level	Numbers (%)
*Clinical recordings*		
Shape of the umbilical outpouching	Elongated	42 (44%)
	Spherical	53 (56%)
Is the content of the umbilical outpouching reducible?	Yes	58 (61%)
	No	37 (39%)
Location of the wound	Ventral	81 (85%)
	Not ventral	14 (15%)
Is the wound superficial?	Yes	80 (84%)
	No	15 (16%)
Is the wound freely manipulable over the underlying tissue?	Yes	79 (83%)
	No	16 (17%)
Is granulation tissue present?	Yes	76 (80%)
	No	19 (20%)
*Macroscopic pathological recordings*		
Morphology of umbilical outpouchings	Hernia	67 (71%)
	Enterocystoma	13 (14%)
	Combination	15 (15%)

**Fig. 2:**
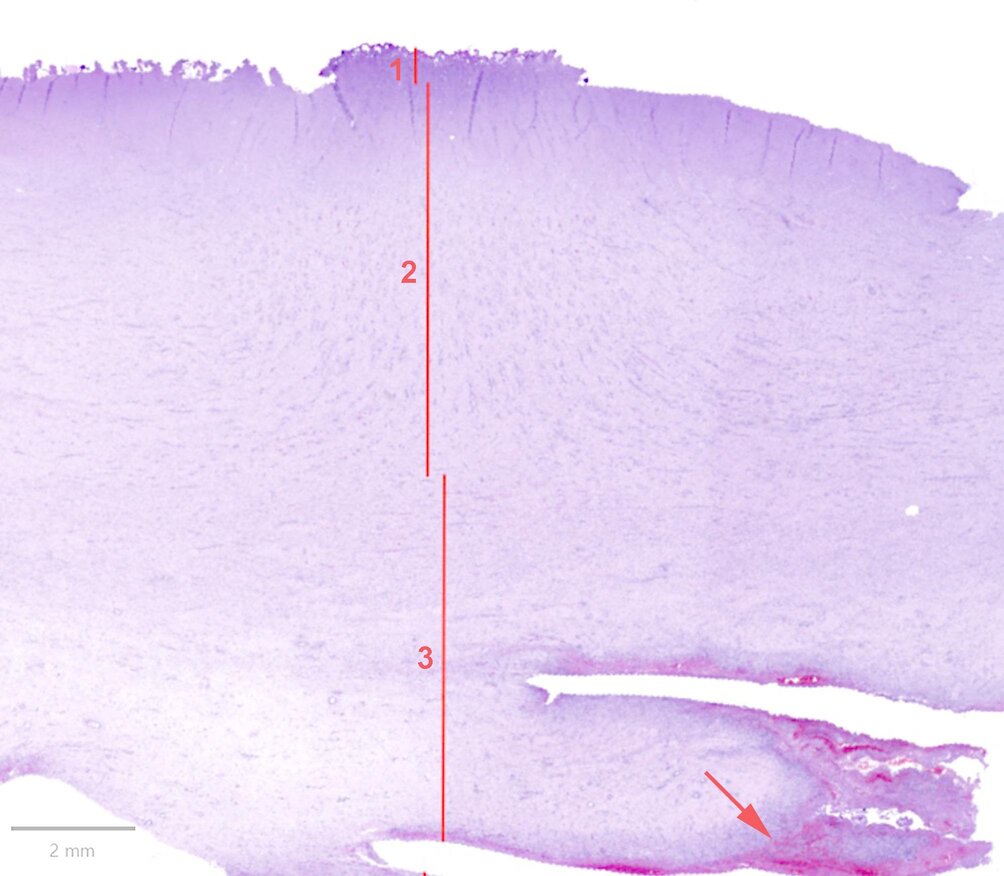
Cross-section of a wound on a hernia. (1) The surface partly covered by a necrotic scab. (2) Underlying wound-associated superficial granulation tissue (S-GT) with perpendicular running vessels. (3) Profound fibrotic tissue (P-FT) with parallel running vessels. Arrow: Hemorrhage

At necropsy, the majority of UOs were recorded as hernias (71%)—and the rest was either recorded as enterocystomas (14%) or a combination of hernias and enterocystomas (15%) (Table 1). A total of 93 UO wounds could be evaluated histologically. The thickness of the scab, S-GT and P-FT are presented in Table 2. In 11 samples, the full extent of the granulation tissue could not be determined. In 5 samples, newly formed granulation tissue was preset below the P-FT. Hemorrhage was present in 9 samples. An example of a full-size sample is shown in Fig. 2.

**Table 2:** Histological measurements in µm of the wound layers The wound layers are the overlaying scab, the superficial granulation tissue related to the wound and the profound fibrotic tissue related to the wall of the umbilical outpouching

	Scab (µm)	Superficial granulation tissue (µm)	Profound fibrotic tissue (µm)
Interval	[114–1501]	[2776–40798]	[0–16706]
Mean	379	11415	4385
Median	295	9871	3697
Standard deviation	274	6855	3346

### Association between clinical signs and pathological characteristics of UO wounds

**Fig. 3:**
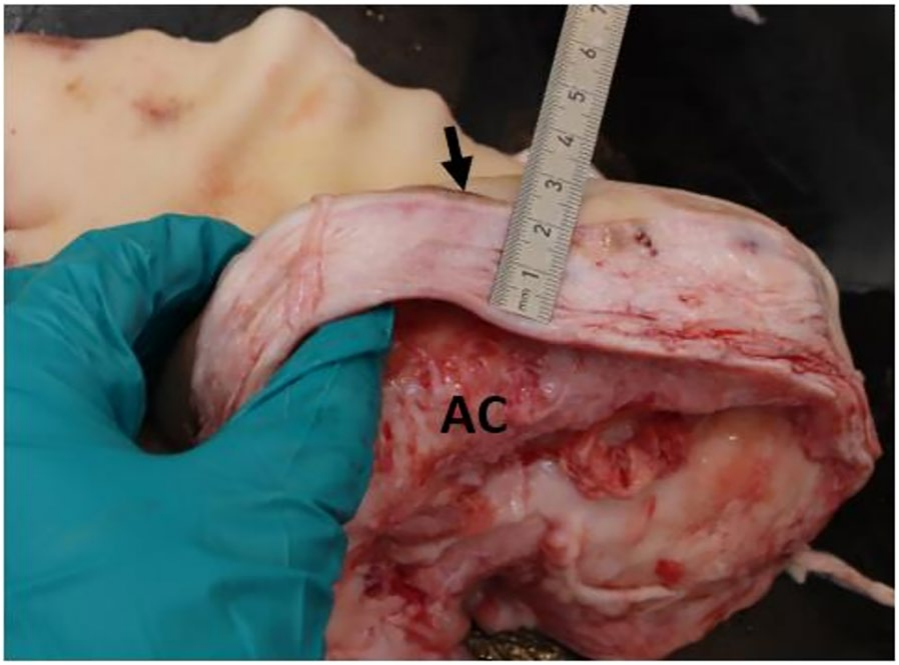
Distance between the wound and the cavity of an umbilical outpouching. Measurement of the distance between the wound (arrow) and the cavity (AC) of the umbilical outpouching

For 8 pigs it was not possible to measure the distance between the surface of the wound and the UO cavity. The mean distance from the surface of the wound to the UO cavity for the 87 pigs was 2.27 cm (σ = 0.89) (Fig. 3).

**Fig. 4:**
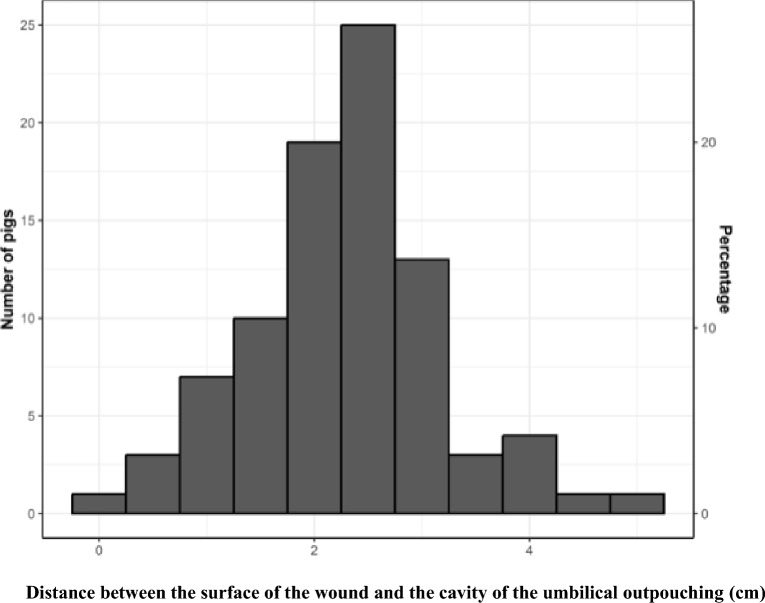
Distance between the wound and the cavity of umbilical outpouching in 87 pigs. Histogram of the distance between the surface of the wound and the cavity of the umbilical outpouching in 87 slaughter pigs

A total of 82 pigs (94%) had a distance of at least 1 cm, 4 pigs (4%) had a distance from 0.5 to 0.9 cm and one pig (1%) had 0 cm, i.e. it had ruptured resulting in access to the UO cavity (Fig. 4).

**Fig. 5:**
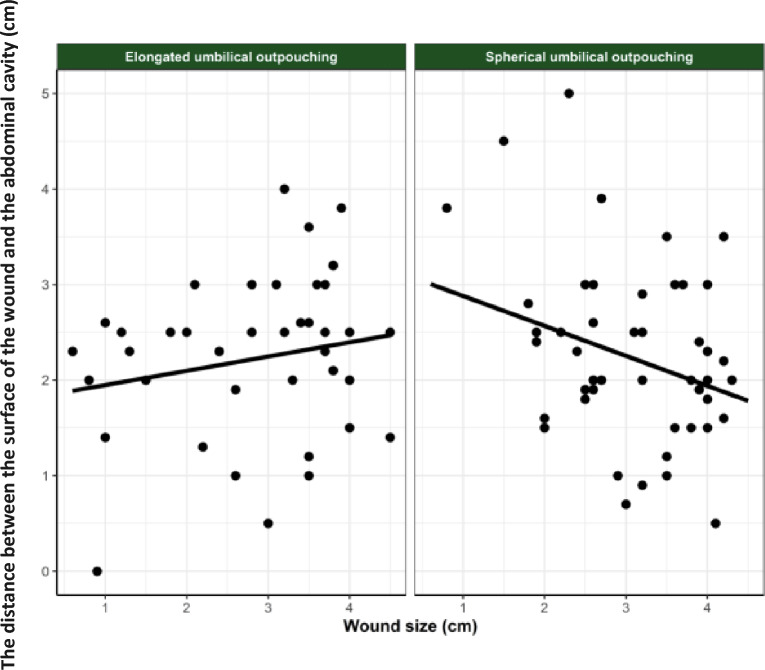
The association between the wound size and the distance between the wound and the cavity. The association between the wound size and the distance between the wound and the cavity of the umbilical outpouching

Results from the multivariable analysis showed no significant effect of the size of the UO and whether the content of the UO was reducible or on the distance from the wound surface to the UO cavity. Moreover, no statistical significance was found for the following clinical signs of the wound in relation to the distance from the wound surface to the UO cavity: localization of the wound, whether the wound was superficial or not, whether the wound was freely moveable over the underlying tissues and if granulation tissue was present. The results showed an association between the size of the wound and the distance from the surface of the wound to the UO cavity which depended on the shape of the UO (Table 3). No statistically significant association was found if the UO was elongated (*P* = 0.22). However, if the UO was spherical the association was significant (*P* = 0.035) —with the distance from the wound surface to the UO cavity being shorter as the wounds became larger (Fig. 5).

**Table 3:** Estimates of the effects of model describing the distance between the wound surface and the cavity

Parameter	Estimate	Standard error	*P* value
Intercept	1.799	0.382	< 0.001
Shape of the umbilical outpouching			
Spherical	1.394	0.580	0.019
Elongated	–	–	–
Elongated*wound size	0.149	0.120	0.22
Spherical *wound size	-0.313	0.146	0.035
Random effect of transport date	0.411		
Residuals	0.778

Data showed that the probability of an UO being a hernia when the shape was elongated was 86% (36/42), whereas the probability of the UO being an enterocystoma or a combination of an enterocystoma and a hernia when the shape was elongated was 14% (6/42). The probability of the UO being a hernia when the shape was spherical was 58% (31/53), whereas the probability of the UO being an enterocystoma or a combination of an enterocystoma and a hernia when the shape was spherical was 42% (22/53).

### Association between sizes of UO wounds measured ante mortem and post mortem.

The mean of the maximum measure of either the length or width of the wound measured clinically was 2.93 cm (σ = 0.97). The corresponding mean wound measures, taken at the abattoir immediately after transport was 2.96 cm (σ = 1.29) and at necropsy 2.87 cm (σ = 1.10).

**Fig. 6:**
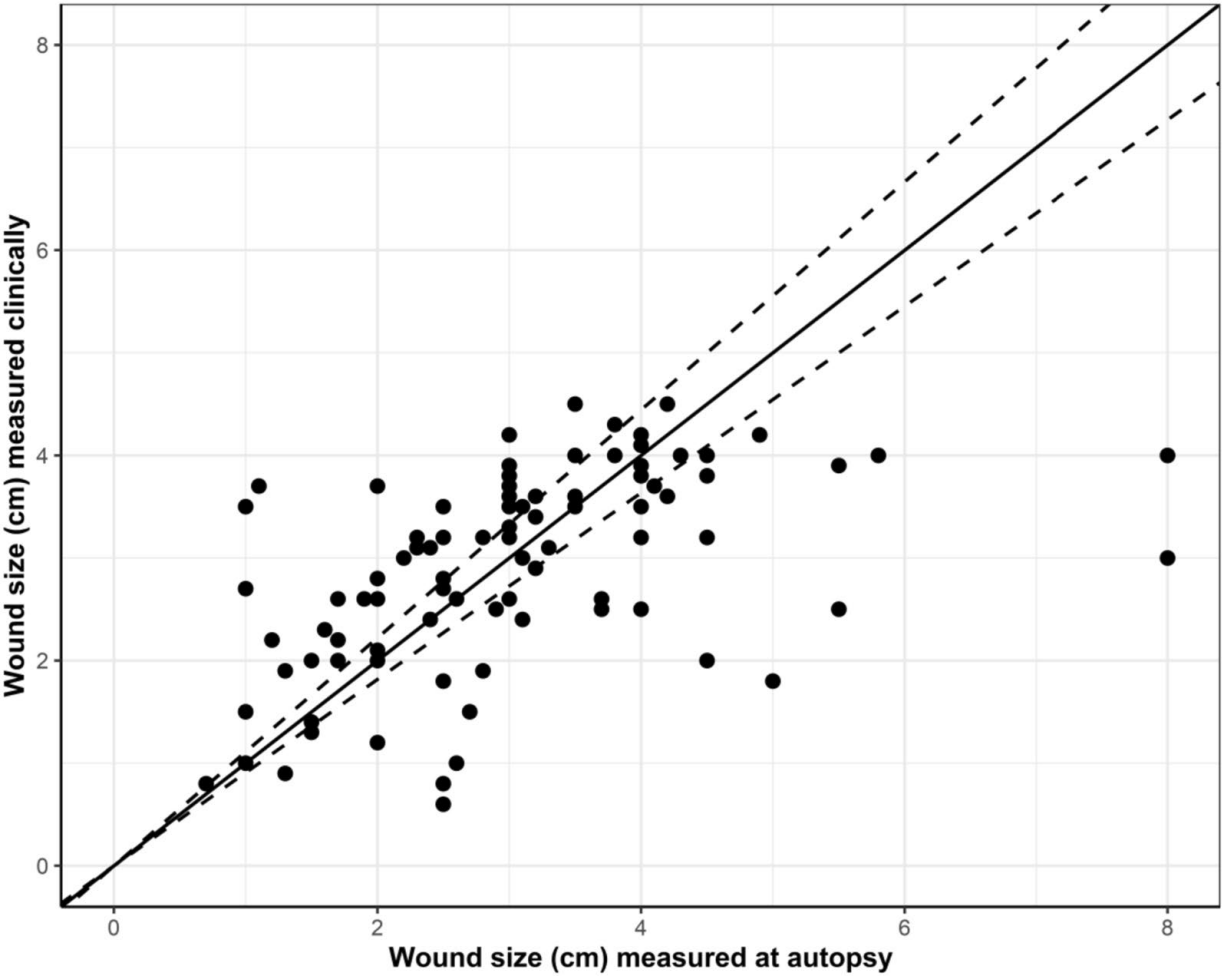
The association between the wound sizes measured clinically and at necropsy. The dotted line represents ± 10% differences from the baseline

When comparing the wound sizes measured clinically and at necropsy, the result showed a Person’s correlations coefficient of 0.51, which can be interpreted as a moderate correlation. The prevalence of wounds measured more than 10% larger at necropsy compared with the clinical examination was 33% (n = 31) and the prevalence of wounds measured more than 10% smaller was 43% (n = 41) (Fig. 6). Wounds on four UOs could not be recorded due to laceration during the slaughter process.

**Fig. 7:**
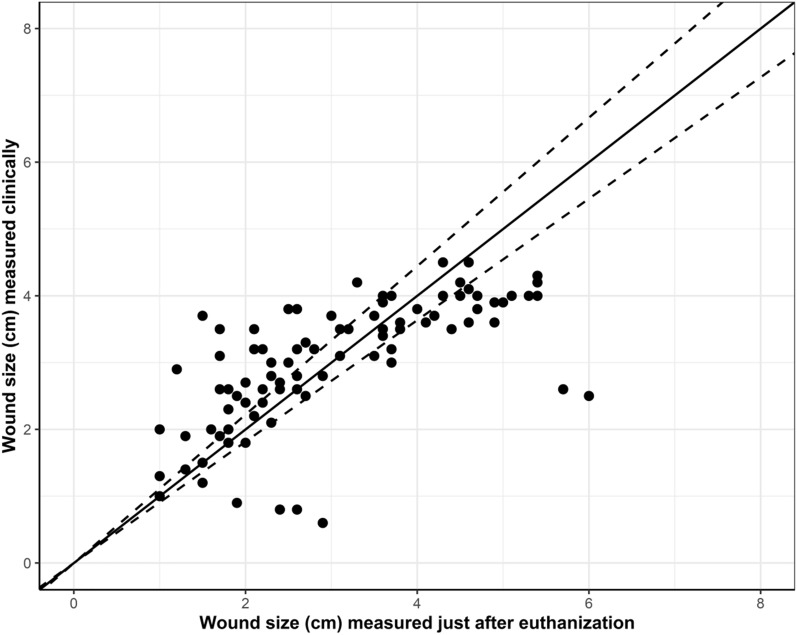
The association between the wound sizes measured clinically and just after slaughter. The dotted line represents ± 10% differences from the baseline

Likewise, when comparing wound sizes measured clinically and just after slaughter at the abattoir, the Pearson’s correlations coefficient was 0.7. This correlation can be interpreted as moderate to high. A total of 30% (n = 28) of the wound measured more than 10% larger at the abattoir compared to the clinical examination and 33% (n = 31) measured more than 10% smaller (Fig. 7).

## Discussion

The potential risk of wound rupture during transport is one of the main reasons why pigs with wounds on UOs are not fit for transport according to the Danish transport regulations. The distance between the wound surface and the cavity of the UO may be used as an indicator of the risk of wound rupture. A shorter distance could indicate a higher risk of rupture. Our study found the mean distance between the wound surface and the cavity of the UOs in 87 slaughter pigs was 2.27 cm, with 94% of the pigs having a distance of at least 1 cm. However, it is important to take into consideration that the substitution of the original skin and underlying tissues with granulation tissue and fibrosis (scar tissue) will lower the original strength of the tissues [12, 13]. The tissue between the surface of the wound and the UO cavity consisted of both fibrotic tissue from the wall of the UO and wound-related granulation tissue. The histological evaluation showed that all wounds were chronic and possessed different amounts of S- GT. The presence of hemorrhage, which likely developed during the transport process in 9 pigs, and newly formed granulation tissue below the fibrous tissue of the UO wall in 5 pigs demonstrated a continuous internal impact on the UOs. This may suggest that these wounds were more vulnerable, potentially leading to a clinical deterioration.

Most wounds were superficial and placed ventrally on the UOs, which has been speculated to be caused from abrasions on the UOs when touching the floor [6]. One wound had ruptured causing access to the UO cavity. This could potentially lead to prolapse of the UO contents. However, in this case, prolapse did not occur. Clinically, this wound was not superficial and placed caudally measuring 0.9 cm in length and 0.8 cm in width.

At the pathological examination, 71% of the UOs were diagnosed as hernias. This agrees with another study examining the morphology of UOs in pigs post mortem [2].

A significant association between the wound size and the distance between the surface of the wound and the UO cavity, that was dependent on the shape of the UO, was found. If the UO was spherical in shape, the distance between the wound surface and the UO cavity and the wound size were inversely correlated. Hansen [14] found that wounds on UOs were most prevalent on enterocystomas. An enterocystoma is a congenital malformation consisting of cysts with fluid filled cavities located intra- and extra abdominally [15]. Clinically, enterocystomas are often hard in texture and are not reducible. The skin upon the enterocystomas is typically stretched. If a wound appears due to abrasions or trauma, the pressure from the enterocystoma might increase the tension on the wound edges. This will lead to reduced micro perfusion causing less oxygen delivered to the wound which consequently will impair wound healing [16]. In the present study, a total of 42% of the UOs that were spherical in shape were diagnosed as enterocystomas or a combination of a hernia and an enterocystoma where the wounds consequently could have had a delay in the healing process due to reduced micro perfusion. The impaired wound healing might have caused the distance between the wound surface and the UO cavity to reduce as the wound increased in size—and hence explain the significant association seen in this study.

On the contrary, no significant association between wound size and the distance between the wound surface and the UO cavity was seen when the UO was elongated in shape. A total of 86% of the UOs that were eloganted in shape were diagnosed as umbilical hernia. Umbilical hernias are due to a defect in closure of the *linea alba* where the umbilical cord passes, and content can pass through the umbilical ring into the hernia sac [17]. Umbilical hernias are usually soft at palpation, and the content may be reducible. The skin upon umbilical hernias is typically not stretched. Due to less pressure of the skin, the conditions for wound healing are more favorable for umbilical hernia, which might explain why no significant association between the wound size and the distance between the wound surface and the UO cavity was found for UOs that were elongated in shape.

Transporting pigs to the abattoir can be challenging for the pigs [18]. Pigs are placed in a new environment and are mixed with other pigs. Although no behavioral data were collected in the present study, it is tempting to speculate that mixing pigs may increase the risk of injuries [4]. This could be due to aggressive behavior from other pigs or injuries associated with the risk of falling when being loaded onto or unloaded from the lorry [19].

Comparing the wound sizes measured clinically and at necropsy we found a moderate correlation of 0.51 between the two measures. The wounds were measured clinically using a ruler placed on the UO of the pig. The examination time was limited due to the pigs’ discomfort with being handled. Additionally, the practical conditions in the pens, such as insufficient light, made the clinical inspection challenging. Therefore, wounds measured clinically may be associated with some uncertainty. Contrary, at necropsy it was possible to measure the size of the wounds precisely. The wounds both measured larger (n = 31) and smaller (n = 41) at necropsy compared to clinically. The reason for wounds being smaller at necropsy was likely due to the presence of scabs which were removed during scalding and dehairing in the slaughter process. The carcasses were hung up by the hind legs on hangers just after slaughter—which made the anatomical appearance different to measuring of the wounds clinically. However, we found a stronger correlation between wounds sizes measured clinically and just after slaughter (0.7) which can be explained by the fact that the pigs had not gone through the slaughter process leaving the wound crust intact.

Wounds measured larger post mortem compared to ante mortem could be due to challenges around transportation of the pigs. In a study of cull sows the clinical conditions deteriorated from farm to slaughter plant—with sows experiencing an increased number of superficial skin lesions [20]. Pigs in our study were placed in separated compartments on the lorry with smaller group size and extra space and soft bedding, which potentially would reduce the risk of the UO and the wound to deteriorate during the short-term transportation to the abattoir. After transportation, the UO wounds of 31 pigs were found to be larger at necropsy compared to the clinical examination.

The Danish Animal Experiments Inspectorate gave permission to transport a maximum of 100 pigs with wounds on the UO where the wounds were maximum 4 cm. Due to the limited number of pigs, we cannot rule out that stronger associations or other associations could be found if more pigs and larger or smaller wounds were included in the study.

## Conclusions

This study is the first of its kind to investigate the association between clinical signs and the pathological characteristics of UO wounds on slaughter pigs following a short-term transportation to abattoir. We found that 94% of 87 pigs examined had a distance from the wound surface to the UO cavity of at least 1 cm, and that one wound had ruptured with access to the UO cavity. For UOs being spherical in shape the distance between the wound surface and the UO cavity decreased as the clinical wound sizes increased. The correlations between wounds measured ante mortem and post mortem were moderate to high—with wounds measuring both smaller and larger post mortem compared to ante mortem. While many factors need to be considered when evaluating fitness for transport, the results from this study may serve as part of a risk assessment for transporting slaughter pigs with wounds on UOs in the future.

## Data Availability

The datasets used and/or analyzed in the study are available upon reasonable request.

## Declarations

### Prior publication

Data included in this article have previously been published in the Proceedings of the 27th International Pig Veterinary Society Congress, Leipzig, Germany, June 4–7 2024.
